# Regulation of the ovarian follicular vasculature

**DOI:** 10.1186/1477-7827-4-18

**Published:** 2006-04-12

**Authors:** Hamish M Fraser

**Affiliations:** 1 MRC Human Reproductive Sciences Unit, The Queen's Medical Research Institute, 47 Little France Crescent, Edinburgh, EH16 4TJ, UK

## Abstract

Angiogenesis is associated with follicular development and is regulated independently within each follicle potentially making the functioning of its vasculature critically important in determining its fate. This review examines the various ways in which follicular angiogenesis may be monitored, describes the follicular localisation and changes in pro- and anti-angiogenic factors that may regulate the process and how antagonists may be used to elucidate their physiological role in vivo. Thus, inhibition of vascular endothelial growth factor (VEGF), VEGF receptor-2, vascular endothelial cell cadherin or interference with the angiopoietin system can inhibit follicular development or prevent ovulation.

## Introduction

Aside from wound healing and certain pathological processes, including neoplasia, the vascular system in the adult is generally quiescent. An exception takes place in the ovaries where there is intense angiogenesis and increased permeability of blood vessels during follicular development, ovulation and subsequent formation of the corpus luteum. Furthermore, angiogenesis is regulated independently within each individual follicle and depending on the extent of the vascular plexus and permeability of vessels, the supply of large molecular weight tropic factors, precursors and lipids can be controlled. This indicates the follicular vasculature could be intimately involved in the processes of follicular selection, dominance and atresia.

It is likely that some types of infertility are associated with disturbance of follicular angiogenesis resulting in inadequate development. In polycystic ovarian syndrome there is excessive angiogenesis while ovarian hyperstimulation syndrome (OHHS) is associated with an increase in capillary permeability. Thus, an understanding of the mechanisms of follicular angiogenesis and its regulation may lead to therapies for controlling inappropriate follicle development secondary to decreased or enhanced angiogenesis. The identification of putative angiogenic factors in the ovary and development of specific agonists or antagonists of angiogenic molecules, together with their application in animal models, presents novel opportunities to validate their physiological role *in vivo*. This review outlines the methods that are being used to study changes in the follicular vasculature, address the work on some of the angiogenic factors which have been studied in the ovary and seem of particular interest at this time, and examines the effects of manipulation of these factors on follicular angiogenesis and development *in vivo*.

### Monitoring of follicular angiogenesis

While primordial and primary follicles receive nutrients and oxygen by passive diffusion from stromal blood vessels, follicular growth is associated with the development of an individual capillary network and continued angiogenesis to nourish the rapidly expanding follicle. The vascular sheath that develops around each follicle is confined to the thecal layer by the presence of the membrana propria until the breakdown of the basement membrane at ovulation. Some of the methods employed to monitor the progress of the follicular vasculature are outlined below.

Measurement of ovarian blood flow can be achieved non-invasively by color and pulsed Doppler ultrasonography in species with sufficiently large and accessible ovaries such as humans, cattle and horses. This demonstrates increased flow to the ovary containing the dominant follicle. In addition, there is increased peak flow velocity with increasing follicular size and high vascularity and flow velocity of the dominant follicle before ovulation [1]. In mares this technique has been used to detect reductions in blood flow area in follicles under conditions where LH stimulation is deficient [2]. New opportunities will arise from advances in technology of high resolution imaging systems for research on small animals, together with the use of contrast agents to enable the imaging of the ovarian vasculature more effectively on a wider scale.

Dynamics of ovarian blood supply to preovulatory follicles has been investigated by injection of radioactive microspheres into the ovarian artery and shows that the elevation in follicular blood supply associated with the preovulatory LH surge is followed by a fall in blood supply as the time of follicular rupture approaches [3]. The spatial distribution of microvessels in the follicular thecal layer may be visualised by scanning electron microscopy of ovarian corrosion casts. This allows angiogenesis to be identified and quantified in individual follicles by identifying sites of budding, sprouting and splitting of capillaries from pre-existing blood vessels. In addition, vascular degeneration can be determined by quantifying numbers of incompletely filled or thinned capillaries [4]. In cattle, angiogenesis was observed mainly in the apical part of the inner capillary layer of medium follicles and the middle or basal part of the capillary layer of healthy dominant follicles. In atretic follicles large avascular areas were observed in the inner thecal layer associated with apoptosis.

The most widely employed approach to study changes in angiogenesis during follicular development is to use ovarian sections in which endothelial cells are stained with a specific marker. Changes in endothelial cell area can then be quantified using image analysis. The most commonly used marker is platelet endothelial cell adhesion molecule (PECAM/CD31), a membrane protein that mediates cell-cell adhesion and is reliably detected in endothelial cells in the follicles of, e.g., the mouse [5,6], rat [7] marmoset [8,9] and macaque [10]. Although CD31 may be used to localise endothelium in the human ovary, CD34, a transmembrane glycoprotein, has proven to be the marker of choice [11,12]. Unfortunately, CD34 antibodies seem to cross-react less in other species. Where neither of these markers are detectable with available antibodies, antibodies to factor VIII/von Willebrand factor may be used successfully to localise the follicular endothelium, e.g. in the mare [13], pig [14] and cow [15]. Recently, another potential marker, vascular endothelial cell cadherin (VE-cadherin), has been localised in mouse follicles [5]. Alternatively, blood vessels may be localised in all species by attachment of carbohydrate lectins that have been biotinylated or fluorescein-labelled as demonstrated e.g., for bovine [15] buffalo [16] and sheep [17] ovaries. In small animals, blood vessels can also potentially be visualised after infusion of lectin [6]. These studies show that large vessels are predominantly located in the outer thecal layer, while smaller vessels are observed in the vicinity of the inner layer close to the lamina basalis. Intravenous injection of labelled gonadotropin in macaques showed highest accumulation in the follicle destined to ovulate [18] suggesting pre-ovulatory follicles had developed the highest blood supply. In most species, vascular area is highest in the dominant follicles [e.g. 9], although in a study of the human ovary, no significant difference in vascular density between dominant and non-dominant follicles were detected [11]. This may reflect species differences in the extent of development of non-dominant follicles. Follicular atresia is associated with inadequate development and/or regression of the thecal vasculature in most species studied [e.g. 8, 9], although this was not confirmed for the human ovary, and it has been suggested that this is related to the longer time taken for the atretic process [11].

Proliferating endothelial cells may be visualised and quantified by dual immunostaining using markers of endothelial cell proteins to localize to the cytoplasm together with proliferation markers localised to the nucleus. For example, nuclear incorporation of exogenous bromodeoxyuridine (BrdU) [8,9] or localisation of Ki67 [10,13] has been widely used. Significant cell proliferation is first seen in the theca of early secondary follicles, when endothelial cell specific staining is still absent, demonstrating that the establishment of the theca compartment precedes angiogenesis [8]. As the follicle continues to develop, endothelial cells are recruited to the thecal layer from the blood vessels in the adjacent ovarian stroma. In the late secondary and tertiary phases of follicular development, 25–30 % of proliferating cells in the inner thecal layer are of endothelial cell origin [8]. The appearance of endothelial cells in secondary follicles with more than 4 granulosa cell layers (late secondary, end of pre-antral stage) [8] coincides with a marked increase in proliferation within the thecal layer. Angiogenesis, calculated by proportion of proliferating endothelial cells, gradually increases in the developing follicle, reaching maximal levels at the late pre-antral/early antral stage. Endothelial cell proliferation is maintained in healthy tertiary follicles to support the ongoing expansion of the thecal vasculature.

In general, the above observations support the idea that vascular development plays a crucial role in the latter stages of follicular growth, and the selection of the ovulatory follicle. In particular, they suggest that efficient elaboration of competent blood vessels and maintenance of vascular permeability is necessary to maintain adequate delivery of gonadotropins, nutrients and oxygen necessary to sustain rapid follicular growth. A disruption of the follicular vasculature during the early stages of atresia would be a logical mechanism leading to a reduced supply of tropic factors and nutrients to the follicle, although such a cause and effect has yet to be established.

### Molecular regulation of follicular angiogenesis

The localisation and regulation of a large number of established and putative angiogenic factors and their receptors in the ovary has been described using a combination of northern blot, RT-PCR and *in situ *hybridization to measure mRNA and western blot and immunocytochemistry to investigate protein. The definition of changes in their temporal and spatial expression patterns in relation to specific stages of follicular development have implied a role for a large number of factors which suggests the process involves a series of complex interactions between numerous regulatory molecules. Although angiogenesis is confined to the thecal layer and the receptors for angiogenic factors are found predominantly on the ovarian vascular endothelium and associated cells, many of the factors that regulate the process are predominantly synthesised in the granulosa.

Studies in which angiogenic factors, or specific antagonists, are administered over selected periods of follicular growth must be conducted in order to confirm the postulated physiological roles of these factors in the regulation of follicular angiogenesis and function. Most studies have employed systemic administration, but the ability to inject factors directly into the ovarian follicle has been also employed [e.g. 19]. Examples of some of the factors that are currently being investigated in the ovary are described below.

#### Vascular endothelial growth factor

Predominant among the candidates is the pro-angiogenic vascular endothelial growth factor (VEGF) family [20]. The most significant of the VEGF is VEGF-A (hereafter VEGF) that is produced in a number of isoforms, VEGF 165 being the most prominent in the ovary [21]. Other members are -B, -C, and D together with placental growth factor (PLGF). VEGF is a potent and specific stimulator of vascular endothelial cell proliferation acting through two tyrosine kinase receptors, VEGFR-1 (formerly known as Flt-1) and VEGFR-2 (KDR). In addition, VEGF has potent permeability actions and may act as a survival factor for immature vessels.

With respect to the localisation of VEGF mRNA and protein during the course of follicular development in the ovary, although there may be some species differences, a general pattern is apparent [9,12]. Typically as in the marmoset, VEGF mRNA is absent from primordial, primary and early secondary follicles and is first detected in the theca and granulosa cell layers of secondary follicles when the follicle develops its own vascular network [9]. Interestingly, this stage of development is largely independent of gonadotropin stimulation and treatment with GnRH antagonist failed to affect VEGF mRNA expression as determined by semi-quantitative *in situ *hybridization [9]. A further increase in VEGF mRNA expression is observed within the granulosa of follicles in the late secondary and tertiary stages, while expression in the theca is lower and remains fairly constant. This is the stage of follicular development that is becoming dependent on gonadotropin stimulation and VEGF mRNA is stimulated by gonadotropins *in vitro *[22] and decreased after treatment with GnRH antagonist *in vivo *[9].

Surprisingly, in follicles that appear very close to ovulation, VEGF synthesis declined in the granulosa cells and exhibited a punctate expression in cells in the thecal layer at the border with the granulosa [8]. The distribution of VEGF receptors in endothelial cells of follicles has been demonstrated by *in situ *hybridization [23] and immunocytochemistry [12].

Specific inhibition of the VEGF pathway *in vivo*, with resultant effects on follicular function, has been achieved using neutralising antibodies against VEGF itself [24], antibodies against VEGFR-2 [25], a VEGFR tyrosine kinase inhibitor [26], the soluble VEGF receptor, sFlt [27,28] and receptor-based antagonists, the VEGF Traps [7,23].

**Figure 1:**
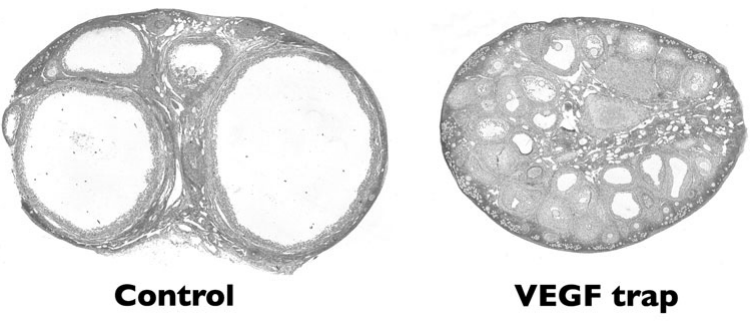
Effect of treatment of marmosets with VEGF Trap on follicular development. Haematoxylin and eosin-stained sections of ovaries of animals treated throughout the 10-day follicular phase with vehicle or VEGF Trap. Note the presence of two healthy dominant follicles in the control ovary, while there is a suppression in follicular development after VEGF Trap. Adapted from [23].

**Figure 2:**
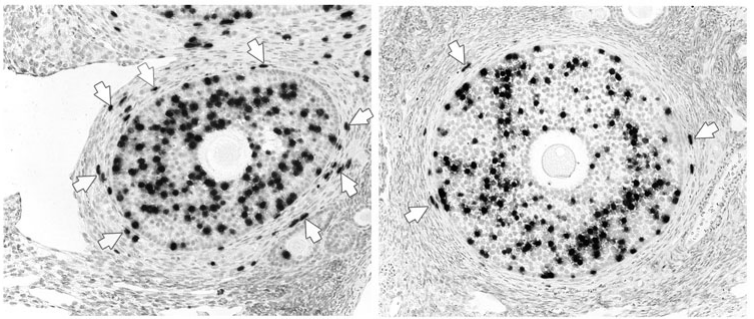
Effects of treatment of marmosets with VEGF Trap for 10 days on thecal and granulosa cell proliferation (BrdU incorporation) in late secondary follicles. Note the high frequency of cell proliferation (arrows) in the thecal layer from control ovary (left panel) with much reduced frequency in the ovary from the treated animal (right panel). This indicates a suppression of endothelial cell and thecal cell proliferation. At this stage of development, there is no effect upon granulosa cell proliferation. Adapted from [23].

VEGF Trap R1R2 comprises Ig domain 2 of VEGF-R1 and Ig domain 3 of VEGF-R2, fused with human Fc. It blocks all isoforms of VEGF A, VEGF B and PlGF but does not inhibit VEGF C and D. Marmosets treated with VEGF Trap from the beginning of the 10-day follicular phase had healthy pre-antral and small antral follicles in their ovaries, but large antral follicles, characteristically present in ovaries of late follicular phase control marmosets, were absent (Figure 1). Morphometric analysis of the theca of late secondary and tertiary follicles revealed a marked decrease in cell proliferation and reduction in endothelial cell area in the treated animals (Figure 2). Cellular proliferation in the theca was reduced by 80% relative to controls, indicating that the proliferation of non-endothelial cells was also inhibited [23]. Antral follicular development was further compromised as indicated by a decrease in granulosa cell proliferation, reduced average diameter of early antral follicles as well as the absence of large tertiary follicles. Expression of VEGFR-1 and VEGFR-2 mRNA in the endothelial cells of the residual thecal vasculature was markedly reduced [23]. As the expression of VEGF receptors is positively regulated by exposure to ligand, this observation provides evidence that VEGF Trap effectively blocked the stimulation of ovarian endothelial cells by endogenous VEGF.

Taken together, the above observations indicate that the inhibition of follicular maturation by blocking VEGF is secondary to attenuation of follicular vascular density and/or permeability, which in turn reduces the availability of growth factors, hormones, lipoproteins, nutrients and oxygen to the growing follicles. These data serve to highlight the absolute requirement for VEGF during follicular angiogenesis and development.

To evaluate the importance of VEGF within the peri-ovulatory follicle in the rhesus monkey, the approach of direct injection of VEGF antagonist (soluble receptor) into the pre-ovulatory follicle has been investigated [29]. Half the animals failed to ovulate but exhibited luteinized unruptured follicles while those which did ovulate had suppressed progesterone secretion with a luteal phase of normal duration.

It is still not established whether the permeability-enhancing actions of VEGF are involved in gonadotropin access to the dominant follicle [18], the development of follicular fluid or the final process of ovulation [30]. Studies to test the hypothesis that expansion of the selected follicle was dependent upon VEGF-mediated permeability examined the effects of treatment of marmosets with VEGF Trap at the mid-follicular phase, the period of follicle selection [31]. However, examination of the most mature follicles 5 days later at the ovulatory period in controls, showed that while angiogenesis had been inhibited, the follicles had undergone expansion suggesting that other mechanisms could compensate for the inhibition of VEGF, or that it is not the primary mediator of follicle expansion.

The effects of inhibition of VEGF at the early, mid- or late follicular phase on pituitary-ovarian hormone profiles in the blood have been described in macaques. When an antibody to VEGFR-2 was administered starting during the early follicular phase, marked changes in pituitary-ovarian function were observed [25]. The follicular phase rise in estradiol was delayed and inhibin B secretion was notably suppressed. This was accompanied by a rapid rise in serum LH and FSH secretion. These results demonstrate the crucial role specifically of VEGFR-2 in follicular development.

**Figure 3:**
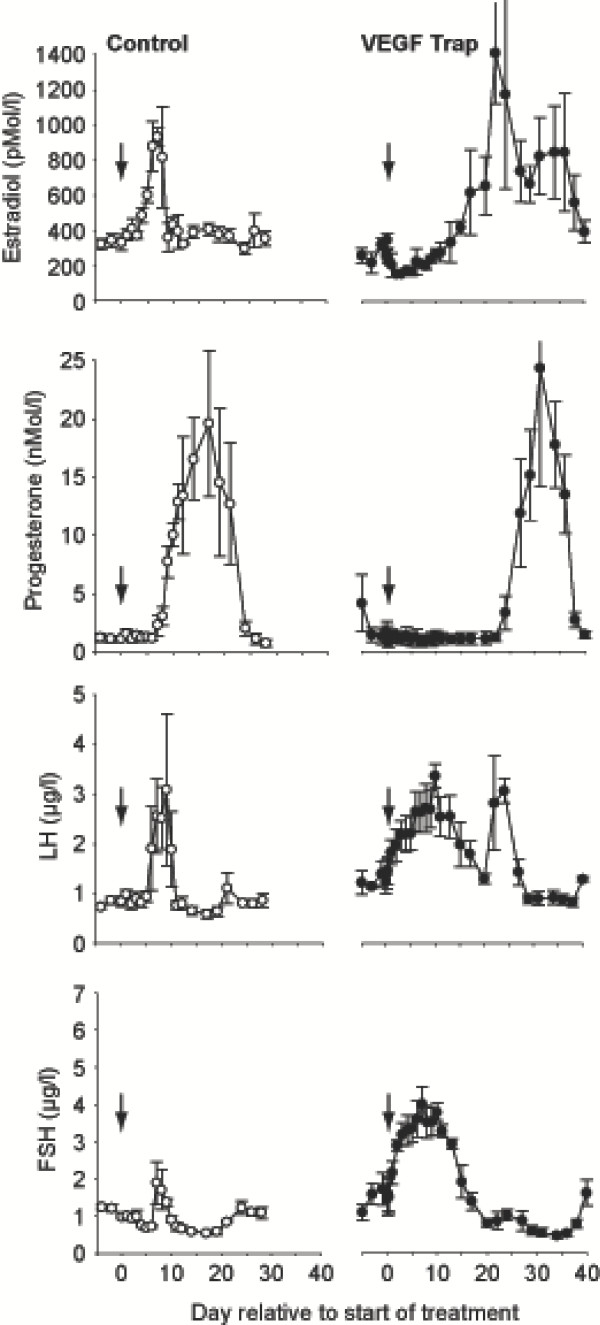
Serum concentrations of estradiol, progesterone, LH and FSH after treatment with vehicle (arrow, open circles) or 0.25 mg/kg VEGF Trap (arrow, closed circles) in the mid-follicular phase in the macaque. Note the timely rise in estradiol, followed by the preovulatory LH/FSH surge and sustained rise in progesterone in controls. VEGF Trap treatment results in a suppression in estradiol and an elevation in LH and FSH but absence of the progesterone rise in the treatment cycle. The progesterone rise indicating ovulation post-treatment took place at 24 days, suggesting recruitment of new follicles was required. Adapted from [32].

Studies with VEGF Trap in the stump-tailed macaque evaluated the hormonal response to a single injection of either 4, 1 or 0.25 mg/kg, i.v. administered during the mid-follicular phase, when the ovulatory follicle is being selected [32]. The VEGF Trap produced a consistent, rapid and prolonged reduction in plasma estradiol and inhibin B levels at all doses, followed by a marked and sustained increase in LH and FSH (e.g., Figure 3). Ovulation took place 7 days after control injections, but was delayed in a dose dependent manner by VEGF Trap. After decline in serum levels of VEGF Trap, the normalization of gonadotropin levels was preceded by an increase in circulating inhibin B and estradiol, and normal post-treatment cycles. These results indicate that inhibition of VEGF at the mid-follicular phase caused atresia of the most developed follicles followed by a dose-dependent period of ovarian quiescence leading to a characteristic follicular phase reflecting the recruitment of a new wave of antral follicles and maturation of a dominant follicle.

When VEGF Trap was administered in the late follicular phase, the anticipated pre-ovulatory rise in estradiol was blocked and although this was followed by a rapid and sustained rise in LH and FSH, there was an absence of progesterone rises which occurred in controls a few days after injection of control protein [32]. Subsequent ovulation was delayed until 32 days after VEGF Trap administration. This suggests that ovulation of the dominant follicle was prevented and a new cohort of follicles were required to be recruited. Administration of an antibody to VEGF during the late follicular phase in the rhesus monkey [24] interrupted the expected late follicular phase rise in estradiol and the follicular phase was extended by a week. This suggested that VEGF inhibition interfered with the latter sages of follicular maturation, but that the follicle remained in a suspended state and could recover from short-term deprivation of VEGF. The more pronounced effects of VEGF Trap at this time may reflect higher dose or potency than the antibody.

All the studies in which VEGF has been inhibited in macaques have resulted in hypersecretion of LH and FSH without apparent stimulation of follicular development. This phenomenon has been studied directly in the mouse. Exogenous gonadotropin administered to hypohysectomized animals stimulated follicle development in controls, but had no effect when VEGF was inhibited using a VEGFR-2-blocking antibody [7]. This supports the idea that VEGF inhibition prevents access of the gonadotropins to the follicles.

It may be that administration of angiogenic factors would be of benefit in cases of ovarian dysfunction characterized by impairments in follicular development and ovulation. Whether administration of VEGF itself might be beneficial in situations where follicular angiogenesis is inadequate is now being addressed. Injection of VEGF gene fragments into the ovarian medulla in pre-pubertal gilts increased VEGF concentration in follicular fluid, follicular vascular density, and the number of pre-ovulatory follicles when given together with gonadotrophin [33]. Treatment of rats with VEGF directly into the ovarian bursa stimulated preantral follicle growth [34], suggesting a role in regulating growth of early follicles. Intra follicular injection of VEGF in the mare resulted in alteration of levels of other factors in follicular fluid but did not increase follicular diameter [19]. Presumably, intra follicular injection of angiogenic molecules will cause them to cross the basement membrane as it is generally acknowledged that the expression of angiogenic factors produced by granulosa and theca cells, acting via endothelial cell receptors, is the principal mechanism by which pro-angiogenic factors promote follicular growth. However, *in vitro *studies suggest angiogenic factors may also act on granulosa or thecal cells directly, in an autocrine or paracrine manner and VEGF receptors have been reported in bovine granulosa cells, suggesting VEGF also has a survival role in non-vascular cells [35].

#### Angiopoietins

While VEGF is the prime initiator of angiogenesis, the formation and differentiation of a structurally and functionally mature vascular network probably requires the co-ordinated action of multiple factors. These include angiopoietin-1 and -2 (Ang-1 and Ang-2), which act via the tyrosine kinase receptor, Tie-2 [36]. Follicular blood vessels are unusual because they grow and mature quickly during follicular maturation and then regress during atresia. By the nature of their action in other systems, the angiopoietins are of particular interest in this context, because they function to influence the stabilisation of newly formed vasculature, as well as the destabilisation of existing vascular networks. Specifically, Ang-1 activation of Tie-2 enhances the maturation and stability of newly formed blood vessels. Ang-2 also binds to Tie-2, but can act as an endogenous antagonist, blocking Ang-1 mediated receptor phosphorylation. In the presence of VEGF, increased autocrine expression of Ang-2 by the vascular endothelium is associated with angiogenesis, while in the absence of VEGF or other pro-angiogenic factors, its expression is associated with degenerative changes in the vasculature [36]. *In situ *hybridization studies on rat ovaries showed that Ang-2 mRNA is absent from preantral follicles and did not become detectable until the pre-ovulatory stage, where its expression is associated with the thecal blood vessels. Ang-1 is expressed in the thecal layer uniformly during follicular development. A dramatic up regulation of Ang-2 mRNA in the granulosa cells of atretic follicles in association with reduced expression of VEGF was observed [36]. Surprisingly, there are few subsequent reports of *in situ *hybridization studies in the ovary. In the marmoset, Ang-1 mRNA was detected in the theca of tertiary follicles, but at a very low level while Ang-2 mRNA was not detected in follicles, but was present in a subset of stromal vessels [8]. In contrast to the rat, high expression of Ang-2 is not apparent in atretic follicles in the marmoset ovary so that further work is required to ascertain whether species differences exist. In bovine follicles at different stages of maturation in which mRNA was extracted from the thecal and granulosa cell layers and subjected to semi-quantitative PCR, expression for both factors was highest in the thecal layer, while the Tie2 receptor was present only in the thecal layer [37]. Changes in mRNA were not marked, but tended to support the idea that changes Ang-1:Ang-2 ratios in follicles were associated with development and atresia. There do not appear to be reports of immunocytochemical localisation of angiopoietins in follicles, although Tie-2 has been described on follicular endothelium [8].

Long-awaited direct evidence for a role the angiopoietins in the ovary comes from observations in the rhesus monkey where local injection of Ang-2 into the pre-ovulatory follicle blocked ovulation [38] while the same dose of Ang-1 was without effect. It is proposed that the Ang-2 inhibited the action of Ang-1 at the level of their common receptor and that the correct balance between these factors is essential in maintaining the integrity of the pre-ovulatory follicle [38]. However, it is not known whether this was a specific angiolytic effect, the result disruption of tissue remodelling or if the Ang-2 affected the hormone-producing cells directly. It will be of great interest to investigate further the mechanisms involved in this action and the effects of manipulation of the angiopoietins at earlier stages of follicular growth.

#### Endocrine gland VEGF

If expression of VEGF is first detected after the initial recruitment of a vascular network to an individual follicle, it is likely that other pro-and anti-angiogenic factors have a crucial role in regulating the onset of follicular angiogenesis. Endocrine gland VEGF (EG-VEGF), also known as prokineticin-1, is a promising angiogenic factor that is expressed at earlier stages of follicular development. EG-VEGF was identified from the human ovary and proposed to function as a specific angiogenic regulator in steroidogenic tissues [39]. EG-VEGF is structurally unrelated to VEGF and acts via G protein-coupled receptors. EG-VEGF mRNA has been shown to be expressed in the follicles of cattle and women [40,41]. *In situ *hybridization of human ovaries shows the mRNA in granulosa cells of primordial and primary follicles, while in antral follicles expression is highest in the theca, and declines in the granulosa [41]. Since EG-VEGF and VEGF appear to exert similar and additive effects *in vitro*, but are differentially regulated and expressed in broadly complementary patterns in the ovary [39], it has been suggested that they may also cooperate *in vivo *to promote angiogenesis and the induction of the fenestrated vascular phenotype [39]. There appear to be major species differences in the physiology of EG-VEGF as in mice it is not present in the ovary, yet is found in the liver and kidney [42]. With respect to elucidation of the physiological role of EG-VEGF, inhibitors of are currently under development and these may be used to investigate the role of EG-VEGF in early follicular development. Since the latter stages of follicular angiogenesis are VEGF-dependent while follicles at early stages of development do not appear to be adversely affected by VEGF-inhibition [23] it will be of particular interest to determine whether EG-VEGF has a predominant role at this time.

#### Cell adhesion molecules

Although the regulation of follicular vascular permeability has long been supposed to have a major impact on the access of hormones and precursors, there currently exists a dearth of information on its molecular regulation in the ovary and the endocrine and paracrine control of the process is just beginning to be addressed [43]. Molecules that are likely have a role in this process, which in turn may be regulated by VEGF amongst other factors, are those involved in cell-cell adhesion, adherens molecules tight junction proteins, occludins and claudins [43,44]. Endothelial cells express cell type specific trans-membrane adhesion proteins such as VE-cadherin at ahherens junctions and claudin-5 at tight junctions [44]. It has been postulated that in the normal ovary VEGF action may involve disruption of endothelial cell tight junction protein complex formation leading to endothelial cell fenestrations. It has been suggested that the hyperstimulation of this process by excessive VEGF and other factors is an integral part of the process of OHHS [45].

A critical role for VE-cadherin in follicular angiogenesis *in vivo *has been demonstrated in the hypophysectomized mouse. VE-cadherin was inhibited by a neutralising antibody that suppressed gonadotropin-induced follicular angiogenesis and development [5].

#### Natriuretic peptides

Natriuretic peptides are a family of small proteins that modulate salt and water balance as well as vascular tone [46]. It has been proposed that atrial natriuretic peptide acts as an anti-permeability factor by inhibiting the signalling functions of VEGF by preserving endothelial cell tight junction morphology [46]. Recently, atrial natriuretic peptide, which has been detected in the ovary, was found to reduce follicular growth when administered intraperitoneally to mice [47] although the question of effects on angiogenesis or ovarian vascular permeability was not addressed.

#### Connective tissue growth factor (CTGF)

Connective tissue growth factor (CTGF) is a cysteine-rich secretory protein which is a member of the cysteine-rich 61/nephroblastoma-overexpressed family of genes [48]. It has been implicated as a paracrine regulator of cell proliferation, in extra cellular matrix remodelling and is generally pro-angiogenic, although in some systems it has been shown to inhibit VEGF-induced angiogenesis. CTGF mRNA is expressed in the theca and granulosa of the porcine [14] and rodent [49] ovary. Absent from small preantral follicles, CTGF expression is highest in the granulosa of mid-antral stage follicles but declines in pre-ovulatory follicles, being down-regulated during FSH-induced granulosa cell maturation [49]. This pattern suggests a role for CTGF in follicular angiogenesis and during the rapid growth of antral follicles. However, studies on CTGF receptor have yet to be described and its role in the follicular angiogenesis has yet to be established. The specific localisation of CTGF mRNA in endothelial cells and fibroblasts in the corpus luteum further supports a role ovarian angiogenesis [14,50].

#### Thrombospondins

Thrombospondins -1 and -2 are large glycoproteins found in the extracellular matrix and act as autocrine factors. Included in the action of thrombospondins are anti-angiogenic and anti-cell migration properties. They are often produced at a rate inversely proportional to that of VEGF and counteract pro-angiogenic stimuli. Thrombospondin localisation in the ovary has been investigated in the rat [51] and cow [52]. Thrombospondin-1 and its cell surface receptor CD36, are localised in highest amounts in the granulosa cells of small developing follicles in the cow; expression then declines such that it is absent from large follicles [52]. Thrombospondin mRNA and protein in the granulosa lysates and follicular fluid concentrations decline as the follicle develops, which is something similar to CTGF [52]. This pattern of synthesis indicates that thrombospondins are involved in suppressing the development of blood vessels during the early stages of development, helping to restrict follicle growth until a synchronized series of events involving the synthesis of VEGF and its receptors is set in motion. In the bovine follicle, thrombospondin production in granulosa cell is stimulated by FSH, while LH has no effect [52].

#### Discovery of other candidates for follicular angiogenesis by microarrays

Another way of gaining information on the role of follicular fluid and granulosa cells in the regulation of endothelial cell gene expression has been to use microarray technology to compare gene expression between unstimulated human umbilical vein endothelial cells treated with either human follicular fluid or culture medium conditioned by human granulosa cells [53]. On the basis of comparing the relative pro-and anti- angiogenic potential, it was concluded that follicular fluid was predominantly anti-angiogenic and it may provide a molecular shield that prevents premature vascularisation of the pre-ovulatory follicle. Thus, down regulation of pro-angiogenic genes, fibulin-5 and elastin and up regulation of Ang-2 and gro-beta (CXCL2) were observed. There is little information on the chemokine gro-beta in the ovary, but in other systems it enhances monocyte adhesion to endothelial cells and inhibits growth-factor stimulated proliferation.

## Conclusion

Angiogenesis plays a critical role in follicular development. The vasculature proliferates and regresses throughout the lifespan of the follicle, but its importance in governing follicle selection on one hand and the early onset of atresia have yet to be established. Many potential candidates involved in the regulation of angiogenesis, vascular permeability and maturation of the vasculature are present in the follicle. For some of these factors, such as VEGF, their functional role can be established by inhibiting their action *in vivo*. The approach described in this review of identifying potential candidate molecules and inhibiting their action *in vivo *using specific antagonists to determine effects on follicular angiogenesis, development and function should provide a powerful tool to elucidate their physiological role and identify ways of either increasing or decreasing ovarian angiogenesis in the clinic.
